# A deep attention LSTM embedded aggregation network for multiple histopathological images

**DOI:** 10.1371/journal.pone.0287301

**Published:** 2023-06-29

**Authors:** Sunghun Kim, Eunjee Lee

**Affiliations:** 1 Department of Information and Statistics, Chungnam National University, Daejeon, Republic of Korea; 2 Department of Artificial Intelligence, Sungkyunkwan University, Suwon, Republic of Korea; Shanghai Maritime University, CHINA

## Abstract

Recent advancements in computer vision and neural networks have facilitated the medical imaging survival analysis for various medical applications. However, challenges arise when patients have multiple images from multiple lesions, as current deep learning methods provide multiple survival predictions for each patient, complicating result interpretation. To address this issue, we developed a deep learning survival model that can provide accurate predictions at the patient level. We propose a deep attention long short-term memory embedded aggregation network (DALAN) for histopathology images, designed to simultaneously perform feature extraction and aggregation of lesion images. This design enables the model to efficiently learn imaging features from lesions and aggregate lesion-level information to the patient level. DALAN comprises a weight-shared CNN, attention layers, and LSTM layers. The attention layer calculates the significance of each lesion image, while the LSTM layer combines the weighted information to produce an all-encompassing representation of the patient’s lesion data. Our proposed method performed better on both simulated and real data than other competing methods in terms of prediction accuracy. We evaluated DALAN against several naive aggregation methods on simulated and real datasets. Our results showed that DALAN outperformed the competing methods in terms of c-index on the MNIST and Cancer dataset simulations. On the real TCGA dataset, DALAN also achieved a higher c-index of 0.803±0.006 compared to the naive methods and the competing models. Our DALAN effectively aggregates multiple histopathology images, demonstrating a comprehensive survival model using attention and LSTM mechanisms.

## 1. Introduction

Medical imaging, including magnetic resonance, computed tomography, positron emission tomography, ultrasound, and X-ray, is essential for the early detection, diagnosis, monitoring, and treatment of diseases. Trained radiologists analyze medical images through visual inspection, which is time-consuming and can result in disagreements between pathologists. To make image analysis efficient and reliable, computational and statistical methods have been developed, such as traditional machine learning models with manually handcrafted features. In 2012, a convolutional neural network (CNN) [1], a deep learning-based architecture, was used for image classification and gained great attention in the medical field. The ability of a CNN to adaptively learn discriminative patterns from image data indicates its usefulness and applicability to medical image analysis. Given recent advances in computer vision and deep neural networks, it has become even more possible to adapt to challenging applications, such as segmentation, registration, and survival prediction [2–4].

Survival analysis is primarily used in the medical field to identify prognostic markers for mortality or disease recurrence and evaluate the efficacy of treatment [5–7]. To utilize medical imaging in survival predictions, deep learning-based Cox regression models have been proposed [8,9]. Specifically, these models predict the hazard rate for each image at a lesion level in histopathology images. However, aggregating and interpreting the results of these models can be challenging when a patient has multiple lesions. Multiple hazard rates that were predicted for a single patient impede a direct interpretation of the results.

In this study, we propose a deep attention long short-term memory embedded aggregation network (DALAN), a deep learning-based Cox regression model for histopathology images of two types of brain tumors: lower-grade glioma (LGG) and glioblastoma (GBM). Our model integrates the processes of feature extraction from multiple lesions and aggregation in one model to predict patient mortality rate. Our model uses attention-based LSTM embedding for survival analysis of histopathological images. We evaluated and compared its prediction performance with other methods on both real and simulated datasets. Our model successfully predicts the patient-level hazard rate by aggregating lesion-level information with attention-based LSTM embedding. Our study was motivated to aggregate lesion-level survival results to compute image- or patient-level survival results for histopathological images. Our contribution lies in combining convolutional neural networks, attention layers, and LSTM layers to perform feature extraction and aggregation of lesion images simultaneously.

In the related work section, we provide an overview of previous research and related methods in deep learning-based survival analysis for histopathology images. In the Materials and Methods section, we detail DALAN and the relevant background knowledge of the method and describe the characteristics of the dataset. In the Results section, we apply DALAN to simulated datasets and The Cancer Genome Atlas (TCGA) to real brain tumor histopathology datasets to evaluate patient-level survival and analyze the results. Finally, we summarize our study and discuss the contributions of our findings.

## 2. Literature review

Recently, deep learning-based Cox regression models have been proposed as a promising approach for survival prediction [8,9]. A neural network-based Cox regression approach has been adapted to optimize the Cox negative partial likelihood, and these approaches have shown comparable or superior performance in survival prediction compared with conventional Cox regression models. This method can be particularly useful for analyzing medical imaging data that cannot be effectively interpreted using conventional methods. Zhu et al. proposed a deep convolutional survival model (DeepConvSurv) that combined Cox regression with CNNs to predict survival using regions of interest (ROIs) of lung cancer histology images labeled with patient information [10]. Morbadersany et al. proposed survival convolutional neural networks (SCNNs) using high-power fields (HPFs) from ROIs for the survival prediction of patients diagnosed with glioma [11]. The whole slide histopathological images survival analysis (WSISA) framework was introduced to utilize the discriminative patterns in the whole slide images (WSIs) and predict patients’ survival status by clustering numerous candidate patches from patients’ WSIs. Yao et al. proposed Deep Attention Multiple Instance Survival Learning (DeepAttnMISL), an attention-based aggregation approach for WSI feature learning [12].

The DeepConvSurv predicts the hazard rate for each patch instead of for each patient, which requires a post-processing step to obtain a patient-level prediction. The SCNNs provide patient-level predictions by calculating the median risk from patch-level risks. The WSISA framework calculates a weighted feature based on the relative number of patches in a cluster and predicts the patient-level hazard rate by using Cox regression with a LASSO penalty. The DeepAttnMISL model adopted a K-means clustering approach to aggregate information from patches. This method clusters patches based on deep learning features obtained from a pre-trained model and uses the resulting clusters as multiple inputs.

To tackle the issue of large-scale WSI and patch-level prediction, multiple instance learning (MIL) has become a popular approach to conducting diagnostic analysis as a form of weakly supervised learning. MIL involves dealing with a group of instances where a single label is assigned. In deep learning-based MIL, a common approach is to apply a pooling operation to instance feature embeddings extracted by a CNN. The overall process can be divided into two stages: 1) constructing an instance-level classifier that maps patches to a sequence of embedding vectors, and 2) designing an aggregation network to generate a bag-level feature vector and calculate the prediction result.

The application of MIL with deep learning has been applied to the training and prediction of medical imaging data. For example, Wang et al. developed a recalibrated multi-instance deep learning (RMDL) method for the classification of gastric cancer [13]. Yousefi et al. utilized a combination of MIL and randomized trees for classifying digital breast tomosynthesis images [14]. Liu et al. proposed landmark-based deep MIL for brain disease diagnosis [15].

In the literature, various aggregation methods have been used for histopathology image analysis. Non-trainable aggregation methods, referred to as naive methods, are used to combine lesion-level information to make patient-level survival predictions. These methods include taking the average of patch-level hazard rates to calculate the patient-level hazard rate or selecting the highest or lowest hazard rate among all patches as the patient-level hazard rate. Mobadersany et al. used a risk filtering method to aggregate survival risk, which involved taking the median values of HPFs [11]. Chunduru et al. aggregated patient-level risk by taking the median ROI-level hazard rates [16]. While these methods are simple to implement, they may not be able to capture complex relationships between ROIs or patches and accurately represent the underlying survival function. While trainable aggregation techniques, such as RNN-based [17,18] and attention-based [12,19–24] aggregations, have also been employed.

However, these methods have separate feature extraction and aggregation steps that are not integrated into their models, leading to potential inaccuracies in the risk prediction for an individual patient. Thus, a more integrated and patient-oriented approach is needed for survival prediction in medical imaging data analysis.

## 3. Materials and methods

### 3.1 Dataset

DALAN was validated using two project datasets: TCGA-GBM and TCGA-LGG from the Cancer Genome Atlas (TCGA) data portal. The TCGA is a collection of cancer specimens with relevant clinical information and histopathological WSIs. The TCGA data is open source, and the subject information is not personally identifiable. Thus, this study is exempted from Institutional Review Board (IRB) review. The demographic information is listed in **Table 1**. A total of 769 patients had 1,061 WSIs, from which 1,505 ROIs were generated from diagnostic tissue slide images. The histological candidate ROIs were curated with a size of 1024×1024 from the WSIs and normalized using sparse stain normalization [25] to match all images with standard H&E histology images. These ROIs were manually reviewed to select tumors with representative histological characteristics, and images containing backgrounds, artifacts, and pen marks or those with poor staining were removed. We denote the ROI containing the lesion as the “lesion ROI”. The 1024×1024 ROIs were then resized to 256×256 for training and testing the model. The preprocessed data were obtained from [11].

**Table 1:** Demographic information of the 769 patients.

Gender	Female	321	Grade	II	181
	Male	448		III	205
Age at diagnosis	< 40	238		IV	350
	40–50	155		Unknown	33
	50–60	179	IDH status	IDH wildtype	335
	> 60	197		IDH mutant	362
Censoring	Censored	381		Unknown	72
	Uncensored	388			

### 3.2 Methods

The basic concept of the Deep Attention LSTM Aggregation Network (DALAN) is to merge feature aggregation and survival prediction to facilitate patient-level analysis in histopathological images. Integrating feature extraction and aggregation within the model enables the training of appropriate features for patient-level predictions. The key techniques utilized in DALAN include survival analysis, convolutional neural networks (CNN), long short-term memory (LSTM), attention mechanisms, and multiple-instance learning for patient-level analysis in histopathological image analysis.

In DALAN, survival analysis predicts patient-wise histologic survival, while CNN specifically extracts survival-related histological features from multiple ROIs. Multi-head attention is incorporated within the Attention-LSTM blocks, calculating the significance of each lesion ROI and allowing the model to focus on relevant features from histopathological images. The LSTM is incorporated in the Attention-LSTM blocks to combine weighted information from lesion ROIs, comprehensively representing the patient’s lesion data. This multiple-instance learning enables the aggregation of lesion-level information to predict patient-level hazard rates, enabling the optimization of CNN parameters and yielding more accurate hazard rate predictions.

#### 3.2.1 Survival analysis

For a specific patient, an event of interest occurring or the last follow-up time before leaving the study is called survival time. Assume that the *i*th individual’s survival time *T*_*i*_ and the censoring time *C*_*i*_ are independent of each other. Then, the observation time is *Y*_*i*_ = min(*T*_*i*_, *C*_*i*_), and each observation corresponds to (*Y*_*i*_, *δ*_*i*_), where the indicator is *δ*_*i*_ = 1 when an event occurs and *δ*_*i*_ = 0 when censored. The survival function representing the probability of survival beyond time *t* is

S(t)=P(T>t),0≤t<∞, (1)

and the hazard function representing the instantaneous risk of surviving up to time *t* and dying immediately after *t* is defined as

$$h(t)=\underset{\mathrm{dt}\to 0}{\lim}\frac{P(t\leq T<t+dt|T\geq t)}{dt}.$$ (2)

Statistical survival models, such as the Cox proportional hazard model, have become one of the main approaches for survival analysis. The Cox proportional hazard model assumes that $h(t|x)=h_{0}(t)exp(\beta^{T}x)$, where *β* is the vector of regression parameters, *x* is the covariates, and *h*_0_(*t*) is the baseline hazard. Estimating the weights of the network *θ* is conducted using the negative partial log-likelihood as a loss function, given by

$$l(\beta ,X)=-\sum_{i\in U}({\hat{h}}_{\theta }(x_{i})-log\sum_{j\in R_{i}}exp({\hat{h}}_{\theta }(x_{j}))),$$ (3)

where ${\hat{h}}_{\theta }(x_{i})$ is the predicted hazard function of the *i*th sample, *U* is the set of uncensored samples, and *R*_*i*_ is the at-risk set whose time of death or follow-up is later than *i*. In this study, adaptive moment estimation with the decoupled weight decay (AdamW) [26] optimizer was used to minimize the loss and optimize the whole model parameter via back-propagation. The concordance index (c-index) was used as the metric to evaluate the models’ performance of survival analysis. The c-index indicates how well the model predicts the ordering of times for a specific sample event. The c-index calculation formula is

$$C‐index=\frac{\sum_{i\in U}\left\{\sum_{T_{j}>T_{i}}1_{f_{j}>f_{i}}\right\}}{\sum_{i\in U}\left\{\sum_{T_{j}>T_{i}}1\right\}},$$ (4)

where *T*_*i*_ is the observed survival time of sample *i*, *f*_*i*_ is the predicted survival time of sample *i*, and 1_*a*>*b*_ returns 1 if *a*>*b* is satisfied and 0 otherwise. This formula evaluates the alignment performance between uncensored and censored sample pairs at time *t*. The c-index has a value between 0 and 1; a value close to 1 is interpreted as a good prediction, and a value close to 0.5 is evaluated as a random guess.

#### 3.2.2 Convolutional neural network

Convolutional neural networks (CNNs) [1] are specialized in extracting image features by using convolution operations on image data. The CNN model extracts an activation map with features of the image data by passing the input image through a network consisting of convolutional, activation, and pooling layers. In the convolution layer, filters represented by the matrix move from the upper left edge of the image data, by the stride specified by the sliding window method, and fill the activation map through the convolution operation. A larger number of convolutional layers increase the depth of the activation map, and the pooling layer reduces the horizontal and vertical dimensions of the activation map while maintaining the depth in the middle of multiple convolutional layers.

#### 3.2.3 Long short-term memory

Long short-term memory (LSTM) [27], a type of recurrent neural network (RNN), can take an arbitrary length of sequence as input. It is widely used in many fields, such as natural language processing and speech recognition. RNNs can memorize past information. However, as the length of the input data becomes longer, the learning ability deteriorates; this is called the vanishing gradient problem. LSTM was devised to resolve this issue. It compensates for the limitations of the feedforward model by using the memory structure of the conditional probability model to capture dynamic sequential patterns.

**Fig 1:**
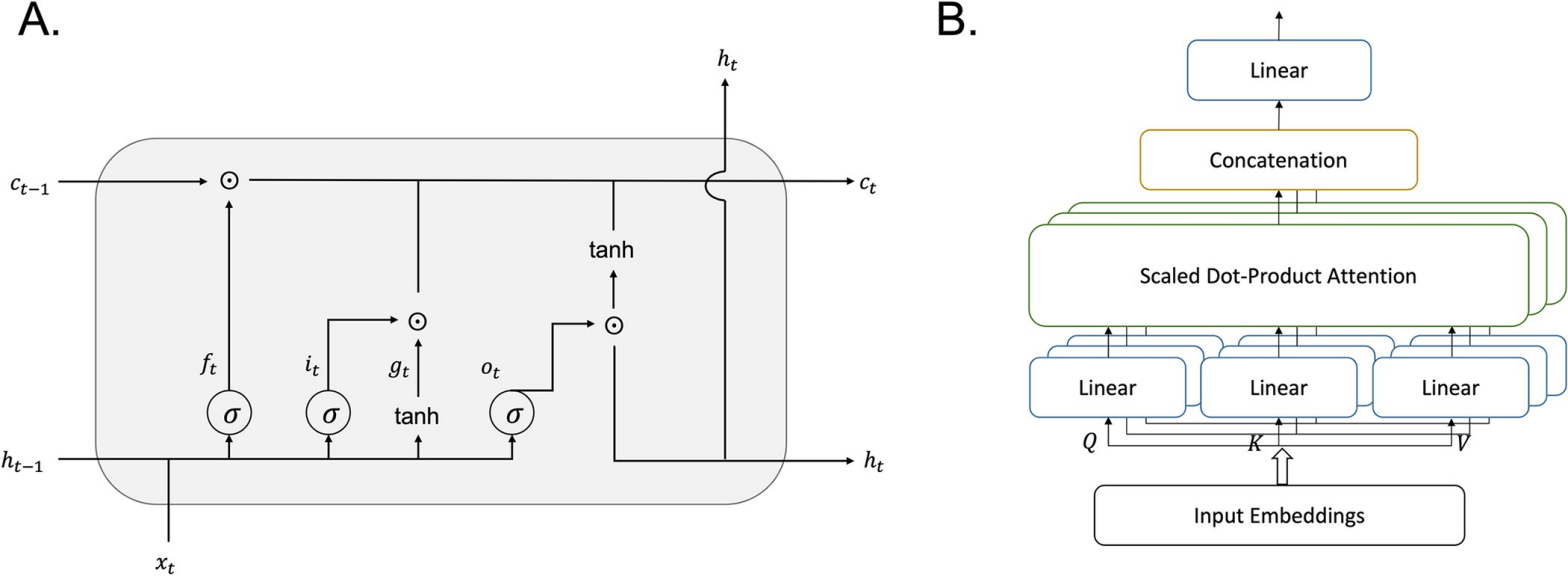
**(A)** LSTM block and **(B)** Multi-head self-attention.


LSTM consists of four gates: the forget *f*_*t*_, input *i*_*t*_, output *o*_*t*_ and input node *g*_*t*_ gates. The state of a memory cell, defined in LSTM as internal memory for storing long-term information, interacts with previous outputs and subsequent inputs to determine the update, retention, or deletion of the internal state. The mathematical expression of the LSTM is given in the following equations, where *σ* represents the sigmoid activation function and ⨀ denotes element-wise multiplication. *W* and *b* are the learning weights and bias, respectively, and, as in the structure of **Fig 1A**, the input *x*_*t*_ at time *t* and the hidden state *h*_*t*−1_ at time *t*−1 pass through several gates to generate the hidden *h*_*t*_ and cell *c*_*t*_ states at time *t* and then propagate to the next time point *t*+1.

$$f_{t}=\sigma (W_{fx}x_{t}+W_{fh}h_{t-1}+b_{f})$$ (5)

$$i_{t}=\sigma (W_{ix}x_{t}+W_{ih}h_{t-1}+b_{i})$$ (6)

$$o_{t}=\sigma (W_{ox}x_{t}+W_{oh}h_{t-1}+b_{o})$$ (7)

$$g_{t}=tanh(W_{gx}x_{t}+W_{gh}h_{t-1}+b_{g})$$ (8)

$$c_{t}=f_{t}⨀c_{t-1}+i_{t}⨀g_{t}$$ (9)

$$h_{t}=o_{t}⨀\tanh\left(c_{t}\right)$$ (10)

#### 3.2.4 Attention mechanism

The attention mechanism [28] has become a widely used deep learning technique in natural language processing (NLP) and speech recognition. This mechanism allows the model to focus on important parts of the input data sequence, and it requires three input variables: query, key, and value. By maintaining contextual information, the attention mechanism captures relationships between elements within a sequence, regardless of their distance. The attention score is determined by a compatibility or distance function, typically the dot-product or cosine similarity, between the query and key. The final weighted expression is obtained by the linear combination of the corresponding attention score and value. When the query, key, and value are all the same, this is called a self-attention mechanism. On the other hand, co-attention uses different embeddings for queries. This approach captures differences between the query and the context, which may improve the performance of the model in NLP tasks. The attention mechanism has a specialized structure for processing time series data and has been used in models that achieve state-of-the-art performance in NLP and speech recognition. The attention map is calculated as follows:

$$Attention(Q,K,V)=softmax\left(\frac{QK^{T}}{\sqrt{d}}\right)V,$$ (11)

where *d* is used to scale the dot-product. The similarity of each pair of key and query embeddings is computed using the dot product of the key and query matrices, which is represented as *QK*^*T*^. Then, a softmax function is applied to this dot product to compute the attention score for each element in the input. This attention score represents the importance of that element in relation to the query. The attention scores are used to compute a weighted sum of the values, where the weight for each value is its corresponding attention score. This weighted sum is then used as the output of the attention layer.

In addition, the attention mechanism can be extended by multi-head attention, as depicted in **Fig 1B**. Multi-head attention is an extension of the attention mechanism that allows a model to attend to different parts of the input in parallel. This is achieved by using multiple attention heads, each of which focuses on a different aspect of the input. Multi-head attention works by transforming the input into multiple queries, keys, and values using linear layers with different parameters. Then, attention is computed for each set of queries, keys, and values to obtain the attention output. The attention outputs from all heads are concatenated along the feature dimension and passed through a linear layer to produce the final output of the multi-head attention layer. By using multiple attention heads, multi-head attention is able to learn different representations of the input data, which can improve the model’s ability to capture complex relationships between the input elements.

#### 3.2.5 Multiple instance learning

Multiple instance learning (MIL) is particularly suited for situations where the data is organized into bags, each containing multiple instances, and where the label information is associated with the bag rather than the individual instances. This type of learning is particularly well-suited for situations where limited information is available about the true labels. One of the main differences in MIL compared to traditional supervised learning is that the labels are not directly assigned to the individual instances. Instead, the label information is associated with the bag, which contains multiple instances.

In traditional supervised learning, a model predicts the value of a target variable *y* based on a single instance. In MIL, however, the input consists of a bag of instances rather than a single instance. The bag of instances *X* = {*x*_1_,⋯,*x*_*K*_} consists of *K* instances that neither exhibit dependency nor order among each other, and *K* could vary for different bags. It is assumed that individual labels exist for each instance within the bag, denoted as *y*_1_,⋯,*y*_*K*_. However, the labels for each instance within the bag are inaccessible and remain unknown. In the context of histopathology images, a bag of instances corresponds to a patient or WSI, while each instance corresponds to patches or ROIs within the WSI.

#### 3.2.6 DALAN

In histopathological image analysis, it is inappropriate to make a prediction of a patient’s hazard rate based on a single patch alone, as a patient may have multiple lesions with distinct ROIs. For patient-level analysis, we propose a deep attention LSTM aggregation network (DALAN) that combines feature aggregation and survival prediction through flexible modeling.

**Fig 2:**
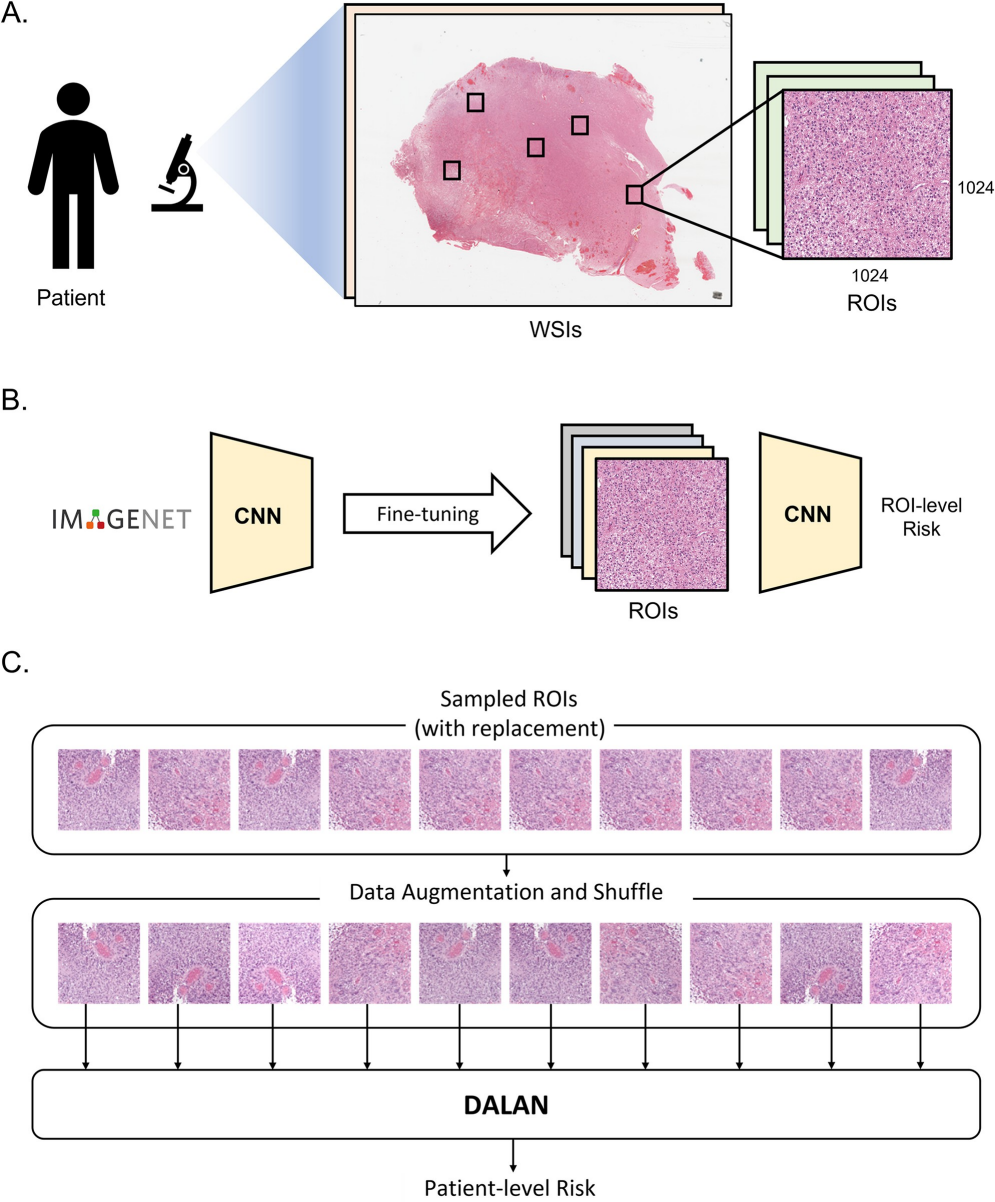
The overall framework. **(A)** The process of identifying representative regions of interest (ROIs) in an image from large whole-slide images (WSIs). **(B)** Fine-tuning the ResNet50 pretrained on the ImageNet dataset via additional ROI-based training. After fine-tuning, CNN weights of half of the front layers are frozen. This CNN is used as a feature extraction network for DALAN. **(C)** The patient’s ROIs are randomly sampled with replacement, and then they undergo data augmentation and shuffling before being fed into DALAN’s input.

The overall framework of our study is presented in **Fig 2**. In this study, each ROI is denoted by a 256×256 RGB three-channel tensor, and the intensity levels of the patch have been scaled to fall between 0 and 1 and then normalized using mean = (0.485, 0.456, 0.406) and std = (0.229, 0.224, 0.225). We used the transfer learning technique to leverage the knowledge gained from a model that has already been trained to solve a related problem. In our study, we utilized a ResNet50 [29] architecture that was pretrained on an ImageNet dataset. However, the weight trained on ImageNet is for natural images, which may differ from histopathology images. To extract survival-related histological features, the CNN of DALAN was additionally fine-tuned for 30 epochs with a learning rate of 1e-5, weight decay of 1e-6, and batch size of 64. This training was based on ROI-level data, where each patient’s survival time was assigned to their respective ROI. To enhance the robustness and prevent overfitting, data augmentation techniques were employed during training, including randomized vertical and horizontal flips and adjustments of brightness and contrast. These fine-tuned CNN weights of the front layers were frozen. Based on these fine-tuned weights, DALAN was trained to predict patient-wise histologic survival by learning an efficient representation of the histologic images.

**Fig 3:**
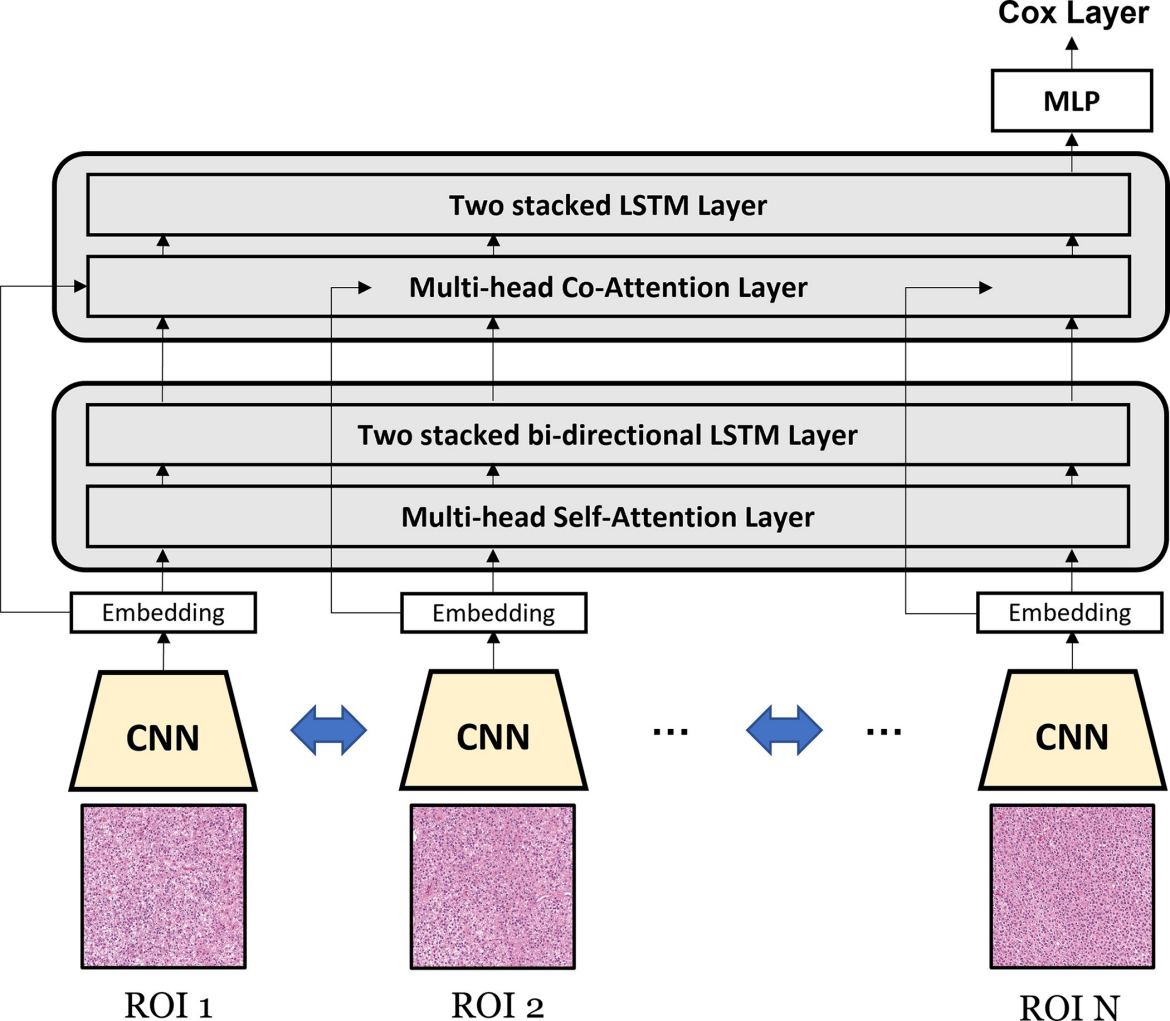
The overall structure of DALAN. The lesion images are compressed into embedding vectors using pretrained CNN and then processed through two Attention-LSTM blocks, which use a multi-head (co-)attention layer and a two-stacked LSTM layer to calculate the significance of each lesion and produce a comprehensive representation of the patient’s lesion data. Cox regression is then performed through an MLP to produce the final hazard prediction. During training, *N* lesion ROIs are randomly sampled, and data augmentation is performed to improve the model’s performance and robustness.

**Fig 3** shows the overall structure of DALAN in detail. The initial CNN weights in DALAN consist of weakly trained ROI-level weights, with half of the layers frozen. This approach is particularly useful when computational resources are limited, and the problem being tackled is similar to the dataset on which the pretrained model was trained. The lesion ROIs are compressed into 256-dimensional embeddings through a weight-shared CNN. The Attention-LSTM block is comprised of a multi-head attention layer with four attention heads and a two-stacked LSTM layer. The lesion ROIs are embedded and then processed through Attention-LSTM blocks, consisting of an attention layer and an LSTM layer. The attention mechanism calculates the significance of each ROI, while the LSTM layers combine the weighted information to produce an all-encompassing representation of the patient’s lesion data. In particular, the second attention layer includes a co-attention mechanism that utilizes the visual embeddings of lesion embeddings as the query. After the information has been processed through two Attention-LSTM blocks, Cox regression is performed using the final sequence of embedding and a multi-layer perceptron (MLP). The last fully connected layer outputs the log hazard in the form of a linear combination.

During both the training and inference phases, we randomly sampled ten lesion ROIs with replacement, and the data augmentation techniques were implemented, including randomized vertical and horizontal flips and transformations of brightness and contrast. This procedure helps to minimize dependence on feature input order and tackles the issue of intra-tumoral heterogeneity. We utilized the gradient clipping technique and optimized the negative partial log-likelihood loss. DALAN was trained using a learning rate of 1e-6, weight decay of 1e-2, a batch size of 32, and 50 epochs. We implemented our model using PyTorch version 2.0, equipped with an NVIDIA RTX 3090 24GB GPU.

A main contribution of DALAN is the model-based aggregation of lesion-level information to predict the patient-level hazard rate. The feature extraction and aggregation are integrated within the model, allowing it to train suitable features for a patient-level prediction. For instance, the patient-level hazard rate can be estimated as the mean of the ROI-wise values predicted by CNN. Unlike this traditional scheme, DALAN also optimizes the parameters of the CNN to make them suitable for patient-wise prediction. This allows for better prediction of the patient-level hazard rate and ensures that the extracted features from the CNN are useful for patient-level survival analysis. Our simulation study provides further evidence of the effectiveness of our method in predicting patient-wise survival.

### 3.3 Simulation study

In this simulation study, we validate our method by adopting a 2D simulated image dataset that is built on a nonlinear survival risk function of imaging information. Our results demonstrate that the performance of our model-based aggregation approach outperforms other naive aggregation methods. To estimate patient-level hazard rates, the simple aggregation methods used the ResNet50 architecture (which is the CNN part of DALAN). The network was trained using Cox negative likelihood loss, and ROI-wise predictions were aggregated using a simple aggregation function. The simple aggregation method adopts a representative value of the ROI estimated hazard rates as the patient-level hazard rate estimate. For example, the average aggregation method calculates the patient-level predicted hazard rate by taking the average value of the ROI-wise predicted hazard rates. Similarly, the minimum and maximum aggregation methods predict the patient-level hazard rate by taking the minimum and maximum values of estimated hazard rates from the ROIs, respectively.

To verify the effectiveness of DALAN’s aggregation method, we conducted two simulation studies using the MNIST survival dataset and the Cancer survival dataset. The MNIST dataset is a commonly used benchmark for image classification algorithms, consisting of handwritten digits, each of size 28×28 pixels. For the MNIST dataset, we used images depicting the digits "0" and "6" as ROIs. On the other hand, the Cancer dataset included the TCGA-LGG and TCGA-GBM datasets, which are also used in analyzing TCGA datasets. Although we utilized the same ROIs in the dataset, we generated survival times without employing the actual patient survival labels. In this manner, we could compare the performance of DALAN to that of the other competing methods in a more realistic setting.

**Fig 4:**
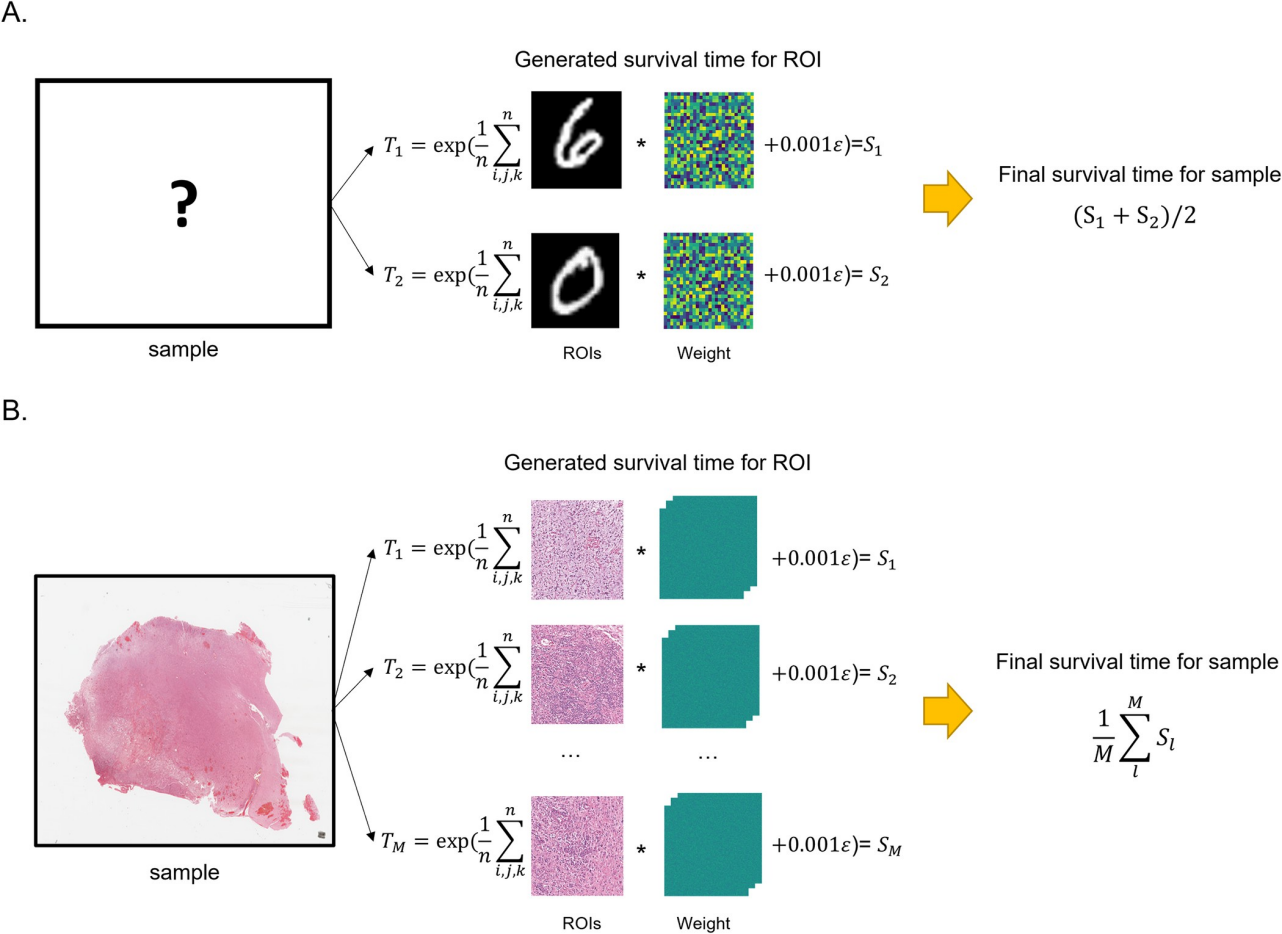
Workflow of the simulation study. **(A)** MNIST dataset simulation. Simulated survival times *T* were generated for two ROIs representing the digits "0" and "6," and these survival times were averaged to obtain the final survival time for the sample. **(B)** Cancer dataset simulation. Likewise, simulated survival times *T* were generated for multiple ROIs, and the final survival time for the sample was obtained by averaging the survival times of all ROIs.

We generated survival times for the images represented by the ROIs in the simulation dataset. **Fig 4** illustrates the workflow of our simulation study. To generate survival times for each ROI, we followed this procedure: first, we generated a random uniform weight $M_{i,j,k}\in R^{\left(W\times H\times D\right)}∼Uniform(0,1)$, where *i*,*j*,*k* denotes the pixel location and *W*, *H*, and *D* denote the width, height, and depth of the image, respectively. We then performed an element-wise multiplication of this weight with each pixel of normalized ROI image *I*_*i*,*j*,*k*_. We randomly censored the final survival times for 50% of the samples. The survival time *T* for each ROI was generated using the following equation:

$$T=exp\left(\frac{1}{n}\sum_{i,j,k}^{n}(I_{i,j,k}*M_{i,j,k})+0.001\varepsilon \right),\varepsilon ∼Lognormal(0,1),$$ (12)

where *n* = *W*×*H*×*D*.

For the MNIST survival dataset, we assumed each sample had two ROIs representing the digits "0" and "6." Each image is represented by an ROI with a size of 28×28, and *T* for each ROI was generated using Eq (12). During the training phase, we randomly sampled 10 ROIs with replacements from the two ROIs. These sampled 10 ROIs are data augmented in the form of randomized flip and rotation and randomly shuffled before being fed into the input of DALAN. Because a subject yields two ROIs, we took the average of the survival times produced for each ROI as the final survival time for the sample.

Similarly, for the Cancer survival dataset, each patient had multiple ROIs. We also generated *T* for each ROI using Eq (12). Each ROI is represented by an image with a size of 1024×1024×3. The final survival time for the sample (i.e., a patient) is then calculated as the average of the survival times across all ROIs. Likewise, we randomly sampled 10 ROIs with replacements during the training phase. After resizing each patch from 1024×1024 to 256×256, these sampled 10 ROIs are then data augmented in the form of randomized flip and brightness and contrast and shuffled randomly before being fed into the input of DALAN. This process leads to a more heterogeneous outcome, and consequently, the final survival time of the simulated data exhibits nonlinearity based on image density information.

Regarding implementation details, the total data were divided into training datasets of 80% and testing datasets of 20%. We repeated the simulation 20 times and finally reported the results. For the MINST survival dataset, both DALAN and the ROI-wise CNN were trained using simple aggregation methods for 100 epochs, with a batch size of 32, a learning rate of 5e-5, a weight decay of 1e-3, and no dropout. For the Cancer survival dataset, DALAN and the ROI-wise CNN were trained for 50 epochs, with a batch size of 32, a learning rate of 1e-5, a weight decay of 1e-3, and no dropout. The learning rate was scheduled with an exponential decay of 0.995 gamma. These hyperparameters were selected empirically, and the models were trained until they reached saturation.

## 4. Results

We validated the effectiveness of the DALAN method in improving the prediction performance of the patient-level hazard rate through simulation studies. To evaluate the patient-level c-index of DALAN, we compared it with competing methods, including simple average, minimum, and maximum aggregation approaches, as well as other competing methods [11,16]. This dataset has been used in previous studies [11,16], and we reported their results. The results of the simulation study and the TCGA analysis are presented in **Tables 2** and **3**, respectively.

**Table 2:** Performance comparison with competing methods on the simulated dataset in terms of c-index (mean±SD).

Methods	MNIST Dataset	Cancer Dataset
Average aggregation	0.901±0.006	0.883±0.009
Min aggregation	0.806±0.009	0.851±0.013
Max aggregation	0.793±0.010	0.849±0.012
Median aggregation [16]	0.900±0.006	0.878±0.008
DALAN	**0.956**±0.004	**0.900**±**0.005**

**Table 3:** Performance comparison with competing methods on TCGA dataset in terms of c-index (mean±SD).

Methods	TCGA Dataset
Average aggregation	0.788±0.009
Min aggregation	0.771±0.015
Max aggregation	0.781±0.013
Mobadersany et al. [11]	0.754
Chunduru et al. [16]	0.790
DALAN	**0.803**±**0.006**

In the MNIST survival dataset, DALAN outperformed other methods with the highest c-index of 0.956. In comparison, the average and median aggregation methods achieved c-index values of 0.901 and 0.900, respectively, while the minimum aggregation method reached a c-index of 0.806 and the maximum aggregation method attained a c-index of 0.793. Similarly, in the Cancer dataset, DALAN again demonstrated superior performance with the highest c-index of 0.900. The average and median aggregation methods obtained c-index values of 0.883 and 0.878, respectively, whereas the minimum and maximum aggregation methods secured c-index values of 0.851 and 0.849, respectively. These results indicate that DALAN offers better performance than simply aggregating information using other methods. Despite the simulated final survival time being generated as the average of the survival times of the ROI-level images, DALAN showed a better result than the average or median aggregation method.

In the TCGA dataset analysis, we compared the performance of DALAN with several competing methods in patients with LGG and GBM brain tumors. The average aggregation method had a c-index of 0.788, while the min and max aggregation methods had c-indices of 0.771 and 0.781, respectively. Mobadersany et al. reported a c-index of 0.754, and Chunduru et al. reported a c-index of 0.790. Notably, DALAN demonstrated the best performance, with a c-index of 0.803.

The integration of crucial elements, including the incorporation of diverse lesion survival information and the optimization of the feature extraction network, enables this achievement. The results reveal that DALAN shows better performance than conventional aggregation methods.

To validate the effectiveness of our DALAN model, we conducted a comprehensive ablation study to identify the significance of each component in the model. The study comprised of several variations of the model, each designed to evaluate the effect of a specific component on the overall performance.

**DALAN w/o fine-tuned weights**: This approach used ImageNet pretrained weights instead of weights fine-tuned at the ROI level. We utilized a batch size of 16, constrained by GPU memory limitations. This allows us to evaluate the impact of weight initialization on the performance of the model.

**DALAN w/o frozen CNN weights**: We evaluated the optimization of the entire CNN weights without partial weight freezing. In this variation, all CNN parameters were optimized.

**DALAN w/o LSTM (only Attention)**: To assess LSTM’s contribution to the aggregation process, the LSTM layer was removed, leaving only two attention layers in the architecture. This variation relied solely on the attention mechanism to focus on important embeddings.

**DALAN w/o Attention (only LSTM)**: To assess the added value of the attention mechanism in emphasizing crucial embeddings, we removed the attention layers, resulting in an architecture with only two LSTM layers. This variation relied solely on the LSTM layers for information aggregation.

**DALAN w/o data augmentation**: We trained the model without data augmentation to measure its impact on the final performance.

The ablation studies help demonstrate the importance of various components in DALAN by showing the impact of their removal on the model’s performance. **Table 4** shows that the full DALAN model achieves the best performance across all datasets, with c-index values of 0.959, 0.900, and 0.803 for the MNIST, Cancer, and TCGA datasets, respectively. Among the ablated versions, the one without data augmentation also performs relatively well but still does not surpass the full model. Both the LSTM and attention components play a crucial role in the overall performance of DALAN. When either component is removed, the performance of the model decreases compared to the full model. The fine-tuned weights enabled the model to generalize more effectively and learn more efficient features for the given tasks.

**Table 4:** Performance comparison with ablation study on simulated and TCGA dataset in terms of c-index (mean±SD).

Methods	MNIST Dataset	Cancer Dataset	TCGA Dataset
DALAN w/o fine-tuned weights	0.954±0.004	0.895±0.007	0.789±0.007
DALAN w/o frozen CNN weights	0.953±0.006	0.891±0.006	0.798±0.006
DALAN w/o LSTM (only Attention)	0.947±0.007	0.892±0.007	0.802±0.008
DALAN w/o Attention (only LSTM)	0.927±0.006	0.881±0.007	0.800±0.006
DALAN w/o data augmentation	0.957±0.003	0.878±0.008	0.798±0.007
DALAN	**0.959**±**0.005**	**0.900**±**0.005**	**0.803**±**0.006**

**Fig 5:**
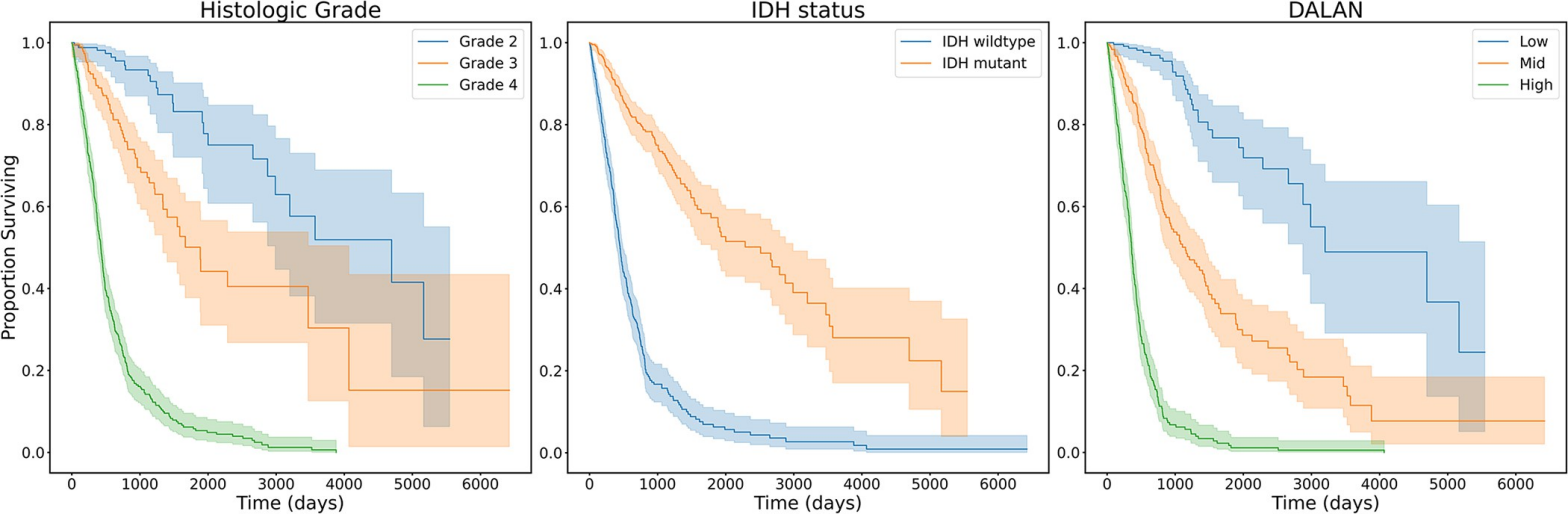
Kaplan-Meier plots according to grade, IDH status, and DALAN.

We analyzed the risk score, which is defined as the log-hazard rates predicted by DALAN, and normalized this score using the mean and standard deviation. **Fig 5** shows the Kaplan-Meier plots of the histologic grading, IDH status, and DALAN. The risk groups are defined as the 0–33 percentile (low risk), 33–66 percentile (mid risk), and 66–100 percentile (high risk) of the predicted risk score. The left and middle plots in the figure show the survival curves based on histologic grade and IDH status, respectively. The median survival times for histologic grades 2, 3, and 4 were 4,695, 1,886, and 435 days, respectively. The median survival times for IDH wildtype and IDH mutant were 448 and 2,988 days, respectively. The right plot in the figure shows the survival curves based on the risk groups stratified by DALAN. The median survival times for the low, mid, and high-risk groups were 4,695, 1,315, and 368 days, respectively. This shows that DALAN effectively stratifies patient outcomes. The high-risk group has a lower survival rate, whereas the low-risk group shows the opposite trend. The log-rank test results also confirm that the survival curves are significantly different (P<1e-4), demonstrating that DALAN is capable of aggregating survival information, dividing patients into distinct treatment groups, and offering personalized therapeutic approaches to each group.

**Fig 6:**
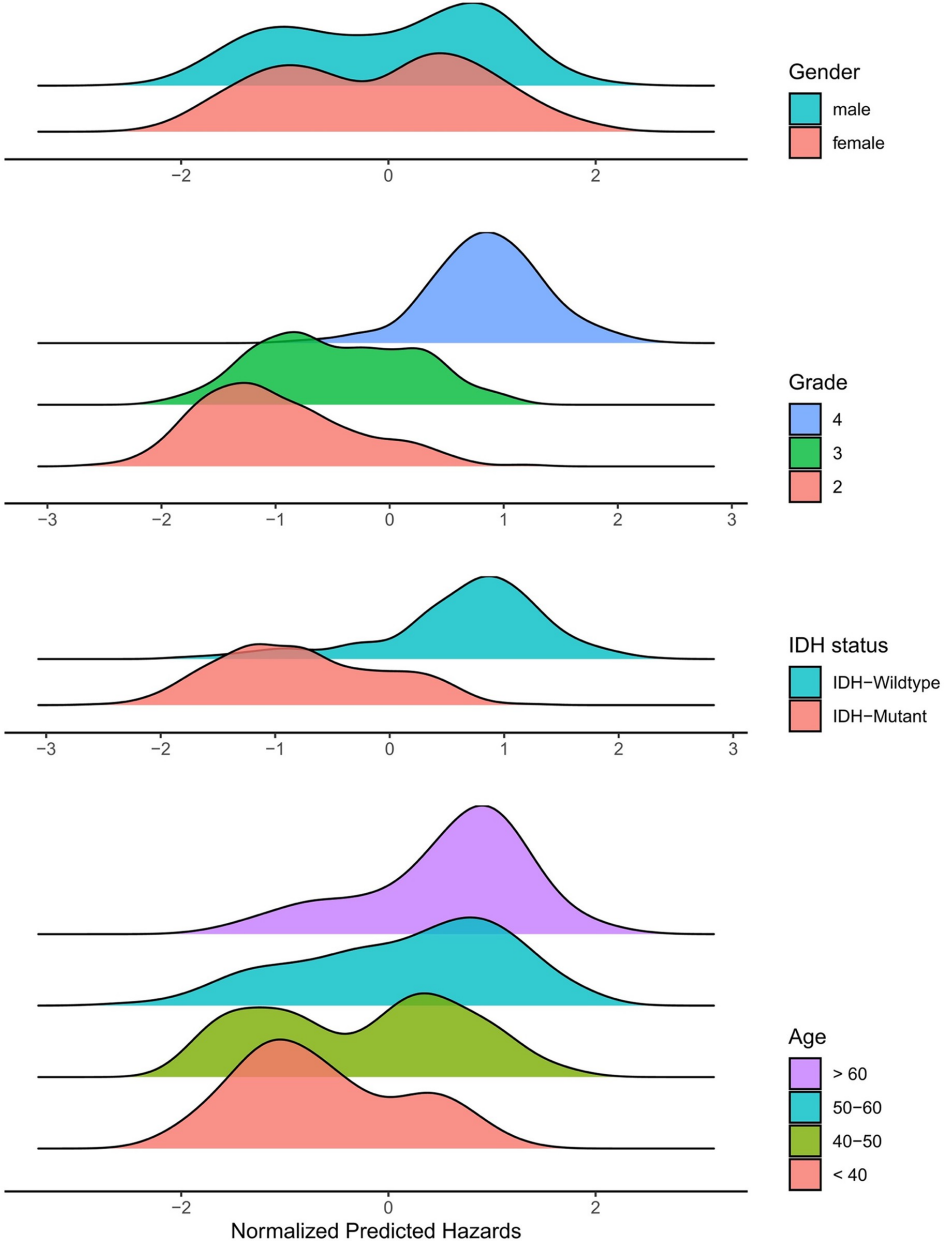
Distribution of normalized predicted risk scores according to gender, grade, molecular IDH status, and age.

We also explored the association between predicted risk scores and various clinical variables. Specifically, we compared the distribution of predicted hazards according to gender, age at diagnosis, histology grade, and IDH status. **Fig 6** shows the ridgeline plots of predicted risk scores according to the clinical parameters.

The results of the analysis of the predicted risk scores are shown in **Table 5**. The p-values were derived from the Wilcoxon rank sum test or the Kruskal-Wallis test. The DALAN predictions exhibited a strong correlation with histologic grades, IDH status, and age and are consistent with the previous studies [30–32]. The results showed no significant difference in the risk score between male and female patients, with a p-value of P = 0.153. However, a significant difference is found in the predicted risk score between the different grades of patients (P<1e-4). Patients with grade 2 had the lowest risk score (-0.993) followed by grade 3 (-0.458), while patients with grade 4 had the highest risk score (0.851). The IDH status of the patients also showed a significant difference in the risk score, with a p-value of P<1-e4. Patients with wild-type IDH had a risk score of 0.65, while patients with mutant IDH had a risk score of -0.75. Age also had a significantly different risk score, with a p-value of P<1e-4. Patients under the age of 40 had the lowest risk score (-0.64), while patients over the age of 60 had the highest risk score (0.56). The risk score for patients between the ages of 40 and 50 was -0.26 and for patients between the ages of 50 and 60 was 0.17.

**Table 5:** Statistical analysis of predicted risk scores.

Clinical Parameters		Mean	SD	Range(min, max)	p-value
Gender	Male	-0.009	1.006	(-2.53, 2.29)	0.153
	Female	-0.108	0.990	(-2.25, 2.03)	
Grade	2	-0.993	0.675	(-2.53, 1.18)	0.000
	3	-0.458	0.675	(-1.99, 1.08)	
	4	0.851	0.495	(-0.87, 2.29)	
IDH status	Wildtype	0.65	0.720	(-1.84, 2.29)	0.000
	Mutant	-0.75	0.720	(-2.53, 1.27)	
Age	< 40	-0.64	0.800	(-2.25, 1.27)	0.000
	40–50	-0.26	0.980	(-1.92, 1.72)	
	50–60	0.17	0.990	(-2.53, 2.03)	
	>60	0.56	0.800	(-1.74, 2.29)	

## 4. Discussion

In this study, we present DALAN, a model-based aggregation method for predicting patient-level survival in brain tumor patients using microscopic images of tissue biopsies. Our approach integrates feature extraction and aggregation steps into one model, leveraging an attention mechanism and LSTM to aggregate lesion-level information into patient-level predictions. The attention mechanism and LSTM in DALAN allow the model to focus on important features and relationships between lesions and capture dynamics in the data. The simultaneous process of feature extraction and aggregation enables the model to efficiently learn imaging features from the cropped lesion images and integrate the lesion-level information to the patient level.

Our simulation study and real data analysis demonstrated that DALAN efficiently learns patient-level imaging features. In the TCGA dataset, DALAN demonstrated a higher c-index of 0.803±0.006, compared to other methods which recorded scores of 0.754 [11] and 0.790 [16]. Additionally, in the simulation study involving both the MNIST and Cancer datasets, DALAN outperformed traditional methods, achieving the highest performance. This can be attributed to the aggregation of survival information from various lesions and the fine-tuning of the image feature extraction network to specific patient-level tasks.

Our main contributions are as follows: DALAN predicts patient-level survival prognosis based on brain tumor histopathology images by considering feature extraction and aggregation within a single model. Our results from simulation and real data analysis showed that DALAN performs better than other competing aggregation methods. In the medical domain, DALAN offers a comprehensive assessment of pathology images, assisting in overcoming the challenge of interpreting various survival predictions for individual patients based on histopathology images.

## 5. Limitation and future work

The proposed method has some limitations. Our study is based on a single data set; the generalizability of our findings should be tested with additional independent datasets in the future. Our current approach is ROI-based, while the trend in the field is toward WSI-based methods. To address this, future work should aim to sample patches with high information content using WSI-based methods. Our approach was confined to imaging data only; thus, a natural future direction could include multimodal data such as genomic and clinical variables.

Moreover, DALAN only deals with right censoring for the observed survival time. Right-censoring occurs when an event of interest occurs after the study period, but it could also happen if an individual withdraws from the study before an event occurs. However, interval censoring is also frequent in cohort studies and clinical trials. Cohort studies and clinical trials measure an endpoint of interest regularly, such as every month or every year, not every day. In those study settings, the true survival time can only be approximated within an interval between two values instead of the exact value. This situation is called interval censoring, which occurs when observing endpoints requires regular follow-ups or thorough inspections. Interval censoring requires imputation or statistical augmentation within the survival analysis. Interval censoring in survival data is a relevant problem in medical settings and has been tackled in several studies using deep learning-based survival models. Meixide et al. exploited the advantages of LassoNet [33] to handle interval censoring and feature selection [34]. Sun and Ding proposed a novel neural network method for interval-censored data (NN-IC) [35]. To enhance the generalizability and robustness of our own models, future work should aim to generalize them to handle these interval censoring conditions.

## 6. Conclusions

We present a deep learning survival model, the Deep Attention Long Short-Term Memory Embedded Aggregation Network (DALAN), that is specifically designed to aggregate survival predictions from multiple lesions at the patient level, thereby addressing the limitations of current methods. In conclusion, DALAN can be used as a comprehensive decision-making tool by radiologists, as it considers multiple lesion images obtained from a patient. Our method could contribute to improved survival prediction of histopathological images by leveraging recent advancements in deep neural networks. Ultimately, DALAN has the potential to help clinicians identify patients with poor prognoses and facilitate prompt treatment, leading to improved patient outcomes. Our work would make a significant step forward in histopathology image analysis and improve patient survival outcomes.
